# Diagnostic Performance of Quantitative Computed Tomography Pulmonary Angiography Parameters in Patients With Pulmonary Embolism

**DOI:** 10.1155/pm/9038741

**Published:** 2025-12-04

**Authors:** Shahab Abdi, Negar Naderi, Seyed Salman Zakariaee

**Affiliations:** ^1^Department of Radiology, Faculty of Medicine, Tabriz University of Medical Sciences, Tabriz, Iran; ^2^Department of Midwifery, Faculty of Nursing and Midwifery, Ilam University of Medical Sciences, Ilam, Iran; ^3^Department of Medical Physics, Faculty of Paramedical Sciences, Ilam University of Medical Sciences, Ilam, Iran

**Keywords:** computed tomography, computed tomography pulmonary angiography, CTPA, pulmonary angiography, pulmonary embolism

## Abstract

**Introduction:**

Pulmonary embolism (PE) is the third leading cause of cardiovascular death after stroke and myocardial infarction. Accurate and timely identification of patients could have a significant impact on reducing the mortality rate and better patient management.

**Aim:**

The purpose of this study was to evaluate the diagnostic performance of quantitative parameters measured based on CTPA images to determine the most important and relevant imaging parameters for diagnosing patients with PE.

**Methods and Materials:**

In this cross-sectional, multicenter study, the electronic files of 1428 cases suspected of PE were reviewed from 2021 to 2023. The diagnostic performances of anthropometric parameters, right ventricle–to–left ventricle (LV) diameter ratio, and CT obstruction index measured based on CTPA images were evaluated for the diagnosis of PTE.

**Results:**

Radiological manifestations associated with PE were IV septum deviation, RV/LV diameter ratio, CT obstruction score, and pulmonary infarction with OR values of 10.53, 38.71, 6.59, and 78.16, respectively (*p* < 0.001). CT obstruction index with a threshold of 1 was the best parameter for the diagnosis of PE. Accuracy, sensitivity, specificity, and AUC of the CT obstruction index were 96.10%, 98.68%, 94.84%, and 0.96%, respectively. Pulmonary infarction with multifocal involvement as the second strongest parameter had a sensitivity of 81.58%, specificity of 98.76%, accuracy of 93.25%, kappa coefficient of 0.93, and an AUC of 0.90.

**Conclusion:**

CT obstruction index and pulmonary infarction with multifocal involvement perform better than the reports of the presence of disease in CTPA images. Therefore, these two parameters must be reported by radiologists and implemented as the primary criteria for diagnosing PE.

## 1. Introduction

Pulmonary embolism (PE) is the third leading cause of cardiovascular death after stroke and myocardial infarction [1–5]. The incidence of PE is approximately 600,000 people per year in the United States, and it causes 100,000 deaths annually in this population [5–7]. The overall mortality rate of PE for untreated patients reaches 20%–35% [6]. With timely disease diagnosis, this mortality rate can be significantly reduced to 2%–10% [8]. On the other hand, exerting unnecessary treatments on patients with suspected embolism can pose irreparable risks to people [9]. Therefore, accurate and timely identification of patients could have a significant impact on reducing the mortality rate and better patient management.

The clinical manifestations of PE (including dyspnea and chest pain) are nonspecific, and they could be observed in other pulmonary or cardiac diseases [9, 10]. In addition, the variable symptoms of the disease, ranging from mild, nonspecific lethargy or breathlessness, to fainting and cardiac arrest, have made the diagnosis of the disease more difficult [11].

Due to the lack of specific signs and symptoms for this disease that accurately confirm the presence of the disease, different diagnostic techniques have been proposed over time. The probability of PE is mainly determined based on the combination of a clinical risk score (e.g., Wells score and revised Geneva score) and D-dimer measurements. These scoring systems were introduced to reduce the number of unnecessary computed tomography pulmonary angiography (CTPA) for diagnosing patients with PE [10, 11]. In this method, if the risk score is low to moderate and the D-dimer result is negative, the possibility of PE is ruled out, and there is no need for further evaluations. However, for patients with a risk score above a certain threshold (e.g., revised Geneva score ≥ 11), the results of the CTPA are used to confirm the presence of PE definitively [10]. The scoring systems are defined based on clinical manifestations, histories, and comorbidities, which led to their average performances.

PE is characterized by embolic occlusion of the pulmonary arterial system [3, 8], and pulmonary angiography is the gold standard method to diagnose PE [2, 12, 13]. However, it is rarely used due to the invasive nature of this procedure and its associated morbidity and mortality rates [12, 13]. In recent years, CTPA has been the method of choice for diagnosing PE. CT is a fast, noninvasive, and widely available imaging method that can scan the entire thorax and directly visualize emboli [4, 6]. The high sensitivity (89%–98%) and specificity (96%–100%) values of the CT approach make it the first-line diagnostic method for patients with suspected PE [1, 3].

In addition to observing emboli on CT images, the severity of the pulmonary arterial tree obstruction could be quantified using the CT obstruction index [14]. In Metafratzi et al.'s study [15], a significant correlation between the degrees of pulmonary artery occlusion quantified using CT obstruction index and blood gas values in patients with PE was reported. Hence, it was suggested that radiologists describe the degree of pulmonary artery occlusion rather than reporting CTPA findings as positive or negative for PE. The performance of CTPA in the diagnosis of PE was determined based on the observation of pulmonary artery occlusion [16, 17]. There is a limited literature investigating the performances of parameters that can be measured based on CT images including anthropometric parameters, right ventricle (RV)–to–left ventricle (LV) diameter ratio, and CT obstruction index for diagnosing PE. The purpose of this study was to evaluate the diagnostic performance of quantitative parameters measured based on CTPA images to determine the most important and relevant imaging parameters for diagnosing patients with PE.

## 2. Methods and Materials

This study was approved by the Institutional Ethics Committee of Ilam University of Medical Sciences (Approved Number IR.MEDILAM.REC.1403.249, approval date: March 11, 2025). To protect the privacy and confidentiality of the patients, the identifying information of the patients was concealed.

### 2.1. Dataset Description

This cross-sectional, multicenter study is aimed at evaluating the diagnostic performance of CTPA parameters in patients with PE. The electronic files of cases suspected of PE were reviewed from 2021 to 2023. The study participants consisted of all people referred to the pulmonary departments of two tertiary care centers in Ilam, Iran. A total of 1428 patients were referred to the pulmonary departments during this period. Demographic information and the definitive diagnosis of the patients were extracted from their hospital records. The definitive diagnosis of PE was made based on clinical signs and symptoms, CTPA data, and D-dimer levels.

In our pulmonary departments, one of the first steps taken for patients suspected of PE is to scan them using the CTPA technique. We used these conventional scanning data to quantify imaging markers. Therefore, during this study, no intervention was made in diagnosing and treating patients, and patients were exposed to no additional risks. The patients with consented to participate and complete CTPA data were included in the study. The exclusion criteria were as follows: (1) patients who did not consent to participate in the study; (2) the patients with other interstitial lung diseases, pulmonary disease, COVID-19, and so forth; (3) the patients with chronic thromboembolic pulmonary hypertension (CTEPH); (4) the patients whose CTPA data were distorted, improperly obtained, incomplete, unreportable, and so forth; and (5) the patients for whom a definitive diagnosis was not available in the file.

### 2.2. Image Acquisition

In this study, two 16-slice multidetector CT scanners were used to scan the patients. The 16 MDCT Brilliance scanner (Philips, Best, Netherlands) used a tube voltage of 120 kV with automatic tube current modulation (80–180 mAs), slice thickness of 1.5 mm, and space between slices of 0.75 mm for patient scanning. The scanning protocol for the 16-slice Aquilion Lightning CT scanner (Canon Medical Systems, Otawara, Japan) was as follows: 100 kV with automatic tube current modulation (80–200 mAs), image reconstruction in 1 mm, slice thicknesses of 1 mm, and space between slices of 1 mm.

The CTPA was performed using a bolus-tracking technique in the pulmonary arterial phase. For this data acquisition technique, 80 mL of contrast agent (Visipaque) with an iodine concentration of 320 mg/mL was injected with a power injector into the patients' antecubital vein. The contrast agent was injected at a rate of 5 mL/s, followed by a 20 mL normal saline flush at the same rate.

### 2.3. Image Analysis

The CTPA images of the patients were retrieved from the picture archiving and communication system (PACS) and blinded for the radiologist. A senior radiologist with more than 10 years of experience reviewed and analyzed the CTPA images on the MIPAV DICOM Viewer (Medical Image Processing, Analysis, and Visualization, Ver. 7.1.1, available at http://mipav.cit.nih.gov/). For each patient, interventricular (IV) septum deviation, pulmonary trunk diameter, diameter of ascending aorta, diameter of azygos vein, diameter of coronary sinus, the degree of contrast material backwash, CT obstruction score, the RV–to–LV diameter ratio (RV/LV ratio), increased RV/LV ratio, pulmonary artery obstruction index (PAOI), and pulmonary infraction were determined based on the CTPA images. Pulmonary artery infarction signs were interpreted as the presence of triangular or wedge-shaped juxtapleural opacifications in areas corresponding to occluded branches of the pulmonary artery. These areas showed hypoenhancement compared to the adjacent normal lung parenchyma and sometimes showed a “halo sign” secondary to adjacent hemorrhage.

The degree of contrast material backwash for the patients was determined as no reflux, reflux trace into the IVC only, IVC involvement only, IVC involvement and involvement of proximal hepatic veins, IVC involvement and involvement of the midportion of hepatic veins, and IVC involvement and involvement of distal hepatic vein subgroups.

The location and extent of pulmonary infarction on CTPA scans were evaluated to classify the patients in pulmonary infarction subgroups. The patients with no pulmonary infarction, unifocal pulmonary infarction, and multifocal pulmonary infarction were scored as 0, 1, and 2, respectively [18].

**Figure 1:**
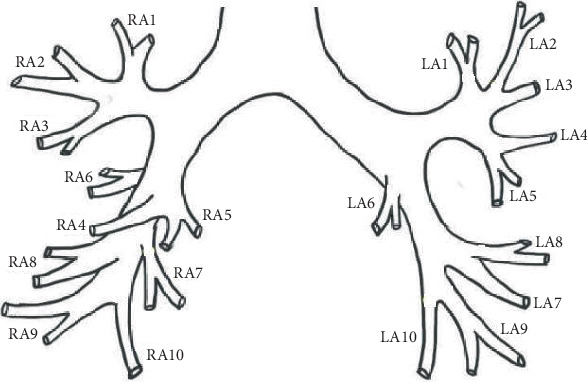
The schematic image used to quantify the CT obstruction score using CTPA images.

The CT obstruction score was calculated according to Qanadli et al.'s method. In this method, 10 segmental arteries, including 5 arteries for the lower lobe, 3 arteries for the upper lobe, and 2 arteries for the middle or lingual lobe, were considered in each lung. These arteries are assigned a score based on their perfusion defect. Arteries with partial and complete occlusion were scored as 1 and 2, respectively. The sum of these scores equals the CT obstruction score, which can range from 0 to 40. The schematic image used to quantify the CT obstruction score is shown in Figure 1.

**Figure 2:**
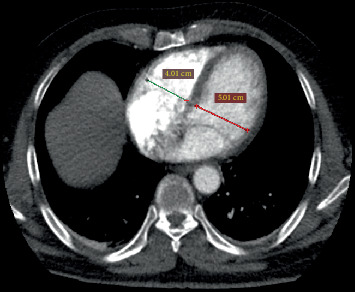
An example of the cross-sectional CTPA image used to measure the right and left ventricle diameters.

The PAOI was determined using the CT obstruction score. For each patient, the PAOI greater and lower than 16 was scored as 2 and 1, respectively [13]. To quantify the RV/LV ratio, the maximum diameter of the RV and LV was measured on CTPA just below the mitral and tricuspid valves, respectively. The right and LV diameters were measured perpendicular to the IV septum on the axial CTPA image (the cross-sectional image with a true four-chambered heart view).  Figure 2 shows an example of the cross-sectional image used to measure the right and left ventricular diameters.

The cutoff point value of 1 was considered the criterion for an increased RV/LV ratio. It means that an RV/LV ratio greater than 1 was classified as an increased RV/LV ratio.

### 2.4. Statistical Analysis

In the descriptive analysis of data, the continuous and categorical data were reported in mean (± SD) and number (*N*), respectively. The significance of the differences in the CTPA parameters between healthy participants and patients with PTE was evaluated at a significance level of 0.01 (*p* < 0.01). The unadjusted and age-adjusted odds ratios (ORs) of the CTPA parameters were determined for the patients with PTE. OR determines the strength of the association between the presence of PTE disease and the CTPA parameters. OR is defined as the ratio of the odds of PTE in the presence of CTPA parameter and the odds of PTE in the absence of CTPA parameter.

The sensitivity, specificity, accuracy, positive predictive value (PPV), and negative predictive value (NPV) indices of the CTPA parameters for the diagnosis of PTE were quantified. The best cutoff point for the continuous variables of the CTPA was determined using the receiver operating characteristic (ROC) curve method.

In this study, the diagnostic performance of quantitative parameters measured based on CTPA images was evaluated for PTE patients. The kappa index (*k*) could determine the agreement between the CTPA parameters and the gold standard diagnosis method. The kappa coefficient is calculated using Equation (1).

(1) $$\begin{array}{c} k=\frac{p\left(a\right)-p\left(e\right)}{1-p\left(e\right)}, \end{array}$$

where *P*(*a*) and *P*(*e*) are the observed and expected agreements between the PTE diagnosis using CTPA parameters and the gold standard method, respectively. *P*(*a*) and *P*(*e*) were calculated as follows:

(2) $$\begin{array}{c} P\left(a\right)=\frac{\text{TP}+\text{TN}}{\text{TP}+\text{TN}+\text{FP}+\text{FN}}, \end{array}$$

(3) $$\begin{array}{c} p\left(e\right)=\frac{\text{TP}+\text{FN}}{\text{TP}+\text{TN}+\text{FP}+\text{FN}}\times \frac{\text{TP}+\text{FP}}{\text{TP}+\text{TN}+\text{FP}+\text{FN}}+\frac{\text{TN}+\text{FP}}{\text{TP}+\text{TN}+\text{FP}+\text{FN}}\times \frac{\text{TN}+\text{FN}}{\text{TP}+\text{TN}+\text{FP}+\text{FN}}, \end{array}$$

where TP, TN, FP, and FN are true positives, true negatives, false positives, and false negatives, respectively. The kappa coefficient ranges from zero to 1. A better agreement between the two diagnosis methods is achieved when the kappa index is closer to 1.

Statistical analysis was performed using SPSS (Ver. 16.0, IBM Corp., United States) and MATLAB (Ver. 2008a, The MathWorks, Natick, Massachusetts, United States) software.

## 3. Results

In this study, hospital records of 1428 people suspected of PTE were retrospectively reviewed. A total of 237 participants remained after applying the exclusion criteria. Based on their medical records, 161 of them were diagnosed as healthy participants and 76 as PTE patients. Seventy-four of the healthy subjects were male, and their mean age was 51.9 ± 16.7 years. The male sex ratio and average age of PTE patients were 51.3% and 51.5 ± 16.1 years, respectively. The descriptive data of the included participants are presented in Table 1. In this table, descriptive data of continuous and categorical variables were separately mentioned for healthy subjects and PTE patients. The significance of the differences in the demographic and CTPA parameters between healthy participants and PTE patients was also listed in the table. The *p* value only indicates the presence or absence of an effect but does not specify the size of that effect. In this study, the concept of effect size was used to assess the statistical strength of the results. The results of evaluating the strength of demographics and CTPA indices for diagnosing PTE patients from healthy subjects are presented in the last column of Table 1. Effect size values of < 0.1, 0.1 to 0.5, 0.5 to 0.7, and 0.7 < were interpreted as no effect, small effect, intermediate effect, and large effect, respectively.

The effect size values indicated that the differences observed for CTPA parameters between PTE patients and healthy participants have significant statistical power and are nonrandom for the sample size studied.

The unadjusted and age-adjusted OR values for demographics and CTPA parameters are listed in Table 2.

The sensitivity, specificity, accuracy, PPV, NPV, and kappa indices of each CTPA parameter for the diagnosis of PTE are reported in Tables 3 and 4. In Table 3, the diagnostic performances of categorical variables of CTPA parameters are presented. For each categorical parameter of CTPA parameters, the sensitivity, specificity, accuracy, PPV, NPV, and kappa indices of each subgroup were separately evaluated.

In Table 4, the diagnostic performances of continuous variables of CTPA parameters are presented. In this table, the best cutoff points with maximum performance for the diagnosis of PTE are listed in the second column.

**Figure 3:**
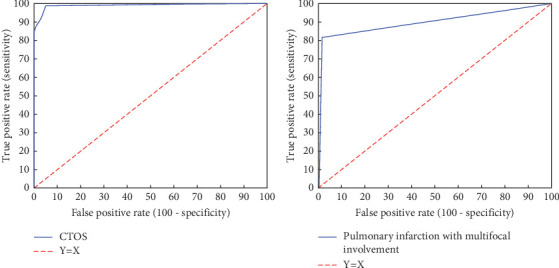
The ROC curves of the CT obstruction score and the multifocal obstruction of the pulmonary arteries as the best CTPA parameters for the diagnosis of PTE.

The ROC curves of the CT obstruction score and the multifocal obstruction of the pulmonary arteries as the best CTPA parameters for the diagnosis of PTE are presented in Figure 3.

## 4. Discussion

Pulmonary vascular resistance would rapidly increase due to PE, which can result in dysfunction of the RV and ultimately lead to heart failure and death [19]. Dysfunction of the RV is associated with a high mortality rate, even if the patients appear clinically stable at first [19]. The high mortality rate of PE causes a lot of costs to society [20, 21].

Therefore, early disease diagnosis is essential to start treatment at the appropriate time.

CTPA has been well accepted as the clinical reference standard for the evaluation of patients suspected of PE. For the diagnosis of patients with PE, the CTPA images are mainly reported as positive or negative for the presence of disease. In a systematic review and meta-analysis study [22], the test accuracy of the CTPA technique in diagnosing suspected patients with PE was evaluated. Their results showed that CTPA, with a sensitivity of 0.94 (95% CI, 0.89–0.97) and specificity of 0.98 (95% CI, 0.97–0.99), has better diagnostic performance than D-dimer, ventilation-perfusion (V/Q) scanning, and compression ultrasonography approaches. These sensitivity and specificity values are the result of merging the sensitivity and specificity values from different studies. In an original research study, the sensitivity and specificity values are much different than these values. In Emet et al.'s study [23], the medical records of 850 patients referred to the emergency department of Hacettepe University Hospital were reviewed between 2001 and 2005. Based on retrospective reviews of these patients, a sensitivity of 95.3%, a specificity of 48.2%, a PPV of 13%, and an NPV of 99.2% were reported for CTPA in the diagnosis of PE.

In recent years, the ability of this imaging method to diagnose PE patients has been reported in terms of diagnostic yield. The diagnostic yield of CTPA for suspected patients with PE in a tertiary care hospital in Saudi Arabia was evaluated by Alshumrani and Mousa [24]. From January 2012 to September 2018, 534 patients with suspected embolism underwent CTPA at this hospital. The probability of PE for the suspected patients was determined based on the Wells score. In this study, radiological manifestations were positive for PE in 177 clinically suspected patients (33.14%). Diagnostic yield only determines the ratio of identified patients using a specific method to all people who were suspected of having the disease and were candidates for undergoing the test. Therefore, the report of the presence of a disease and diagnostic yield cannot present the actual capability of CTPA in the diagnosis of PE. There is a need to investigate the parameters that can be calculated based on CTPA images to determine the most important and relevant imaging marker for diagnosing embolism. In this study, the performances of RV–to–LV diameter ratio, CT obstruction index, and anthropometric parameters that were quantified using CTPA images were evaluated for the diagnosis of PE.

The primary analysis of results showed that, except for age, gender, BMI, and ascending aorta diameter, significant differences were observed for all parameters extracted based on CT images between the two groups of embolic and nonembolic patients (*p* < 0.001).

The convexity of the IV septum in nonembolism subjects was more towards the RV. However, in patients with embolism, the IV septum was significantly flattened or deviated towards the LV due to RV pressure and volume overload (*p* < 0.001). In measurements that were made based on CTPA images, no significant difference was observed for the diameter of the ascending aorta between the embolism and nonembolism subjects (*p* = 0.962). But the diameters of the azygos vein, coronary sinus, and pulmonary artery were significantly greater in patients with embolism than in nonembolism subjects (*p* < 0.001).

The diameter of the pulmonary artery can increase due to several factors, including obstruction and subsequent changes in hemodynamics. The clot physically blocks blood flow, leading to increased pressure in the pulmonary circulation. This elevated pressure, known as pulmonary hypertension, can lead to an increase in the diameter of the pulmonary artery. Additionally, the body's response to the clot, including vasoconstriction (narrowing of blood vessels) in other areas to compensate, can further exacerbate the pressure and dilation of the pulmonary artery.

The body may try to compensate for the reduced blood flow to the lungs by increasing blood flow through other vessels, including the azygos vein, leading to an increase in their diameters. Other reasons for observing an increase in the diameter of the azygos vein might be the increased pulmonary artery pressure, RV strain and failure, function of the azygos vein as a collateral pathway, and so forth.

A dilated coronary sinus is often observed in PTE patients; this increased diameter is frequently associated with impaired RV function. Pressure elevated within the RV, which is a common consequence of PTE, leads to a greater volume of blood flowing into the coronary sinus and increases the diameter of the coronary sinus.

For the RV/LV diameter ratio evaluated between the embolism and nonembolism subjects, a higher ratio was observed in the embolic patients than in nonembolic subjects (0.93 ± 0.26 vs. 0.79 ± 0.15), and this difference was statistically significant between the two groups (*p* < 0.001).

The increase in the RV/LV diameter ratio of the PTE patients indicates RV dysfunction, which is often due to increased pulmonary artery pressure and afterload. The blood clot in the pulmonary arteries restricts blood flow to the lungs and makes the RV work harder to pump blood through the narrowed vessels. Consequently, the RV dilates, and its diameter increases relative to the LV, leading to a higher RV/LV diameter ratio.

In quantifying the degree of pulmonary artery occlusion based on CTPA images, the results showed that the obstruction scores and multifocal involvement were significantly higher in patients with embolism (*p* < 0.001). Radiological manifestations associated with PE were IV septum deviation, RV/LV diameter ratio, CT obstruction score, and pulmonary infarction with OR values of 10.53, 38.71, 6.59, and 78.16, respectively.

In the next step, the classification performances of the RV–to–LV diameter ratio, CT obstruction index, and anthropometric parameters for diagnosing PE were evaluated. Results showed that the CT obstruction index with a threshold of 1 is the best parameter for the diagnosis of PE. Accuracy, sensitivity, specificity, and AUC of the CT obstruction index were 96.10%, 98.68%, 94.84%, and 0.96%, respectively. Pulmonary infarction with multifocal involvement was the second strongest parameter for diagnosing PE. The multifocal obstruction of the pulmonary arteries could discriminate patients with PE from nonembolic patients with a sensitivity of 81.58%, specificity of 98.76%, accuracy of 93.25%, kappa coefficient of 0.93, and an AUC of 0.90. These two parameters had excellent performance for identifying patients with PE, with a significant difference in their diagnostic performance compared to other parameters. It must be noted that these two parameters express the same concept because multifocal obstruction of the pulmonary arteries results in a higher magnitude of CT obstruction index.

Comparing the diagnostic performance of the CT obstruction index with the results reported in the literature shows that this parameter performs better than the reports of the presence of disease in CTPA images (the common method of reporting for clinical cases).

These results indicate that while multifocal obstruction of the pulmonary arteries can serve as an additional diagnostic index for PE during the transition period, the CT obstruction index must be reported by radiologists and implemented as the primary criterion for diagnosing PE, as mentioned in Metafratzi et al.'s study [15].

## 5. Conclusion

In this study, the performance of quantitative parameters measured based on CTPA images was evaluated to determine the most important and relevant imaging parameters for diagnosing patients with PE. Results showed that the CT obstruction index with a threshold of 1 and pulmonary infarction with multifocal involvement are the best diagnostic parameters for the diagnosis of PE. Comparing the diagnostic performances of these two parameters with the results reported in the literature shows that these parameters perform better than the reports of the presence of disease in CTPA images. Therefore, CT obstruction index and pulmonary infarction with multifocal involvement must be reported by radiologists and implemented as the primary criteria for diagnosing PE.

## Figures and Tables

**Table 1:** The descriptive data of included participants and the significance of the differences in the CTPA parameters between healthy participants and PTE patients.

**No.**	**Parameter**	**M** **e** **a** **n** ± **SD or frequency in healthy participants (N** = 161**)**	**M** **e** **a** **n** ± **SD or frequency in patients with PTE (N** = 76**)**	**p** ** value**	**Effect size (** **d** **)**
1	Age (year)	51.88 ± 16.71	51.47 ± 16.14	0.888	0.02
2	BMI (kg/m^2^)	23.69 ± 3.36	24.98 ± 5.34	0.418	0.18
3	Sex				
		Female (87) Male (74)	Female (37) Male (39)	0.441	0.10
4	Degree of contrast material backwash				
		No reflux (107) Reflux trace into the IVC only (26) IVC involvement only (7) IVC involvement and involvement of proximal hepatic veins (9) IVC involvement and involvement of the midportion of hepatic veins (2) IVC involvement and involvement of distal hepatic veins (0)	No reflux (26) Reflux trace into the IVC only (17) IVC involvement only (16) IVC involvement and involvement of proximal hepatic veins (10) IVC involvement and involvement of the midportion of hepatic veins (6) IVC involvement and involvement of distal hepatic veins (1)	< 0.001	0.88
5	IV septum deviation				
		Convex to right ventricle (143) Flattened septum (9) Convex to left ventricle (2)	Convex to right ventricle (33) Flattened septum (35) Convex to left ventricle (8)	< 0.001	1.31
6	Pulmonary artery diameter (mm)	26.77 ± 4.21	28.86 ± 4.21	0.001	0.49
7	Ascending aorta diameter (mm)	32.97 ± 4.54	33.00 ± 5.41	0.962	0.01
8	Azygos vein diameter (mm)	9.17 ± 1.84	10.29 ± 1.96	< 0.001	0.56
9	Coronary sinus diameter (mm)	9.48 ± 2.03	10.98 ± 2.55	< 0.001	0.64
10	RV/LV diameter ratio	0.79 ± 0.15	0.93 ± 0.26	< 0.001	0.52
11	Increased RV/LV ratio				
		Not increased (148) Increased (12)	Not increased (52) Increased (23)	< 0.001	0.64
12	CT obstruction score	0.10 ± 0.45	13.93 ± 9.49	< 0.001	2.62
13	Pulmonary artery obstruction index				
		PAOI < 16 (161) PAOI ≥ 16 (0)	PAOI < 16 (44) PAOI ≥ 16 (32)	< 0.001	1.41
14	Pulmonary infarction				
		No (153) Unifocal (6) Multifocal (2)	No (1) Unifocal (13) Multifocal (62)	< 0.001	5.03

**Table 2:** The unadjusted and age-adjusted OR values for demographics and CTPA parameters for the diagnosis of PTE.

**Parameter**	**OR**				**Adjusted OR**			
	**p** ** value**	**OR**	**95% CI for OR**		**p** ** value**	**OR**	**95% CI for OR**	
			**Lower**	**Upper**			**Lower**	**Upper**
Age	0.862	0.998	0.982	1.016	—	—	—	—
BMI	0.194	1.069	0.967	1.183	0.237	1.064	0.960	1.179
Sex	0.442	1.239	0.718	2.140	0.464	1.235	0.702	2.172
Degree of contrast material backwash	< 0.001	1.976	1.525	2.561	< 0.001	1.981	1.507	2.604
IV septum deviation	< 0.001	10.527	5.211	21.268	< 0.001	10.118	4.947	20.691
Pulmonary artery diameter	0.001	1.121	1.049	1.199	0.001	1.129	1.050	1.215
Ascending aorta diameter	0.962	1.001	0.946	1.060	0.845	0.993	0.924	1.067
Azygos vein diameter	< 0.001	1.361	1.165	1.590	< 0.001	1.363	1.159	1.603
Coronary sinus diameter	< 0.001	1.328	1.152	1.531	< 0.001	1.415	1.205	1.663
RV/LV diameter ratio	< 0.001	38.708	7.650	195.853	< 0.001	56.666	10.194	314.999
Increased RV/LV ratio	< 0.001	1.185	1.098	1.279	< 0.001	1.198	1.107	1.296
CT obstruction score	< 0.001	6.594	3.308	13.145	< 0.001	6.384	3.130	13.022
Pulmonary artery obstruction index	0.997	6e + 9	< 0.001	NM.	0.998	6e + 9	< 0.001	NM.
Pulmonary infraction	< 0.001	78.165	23.267	262.593	< 0.001	71.362	21.485	237.030

*Note:* NM: not measurable (the upper limit of 95% CI for OR cannot be measured).

**Table 3:** The sensitivity, specificity, accuracy, PPV, NPV, kappa, and AUC indices of categorical variables of CTPA parameters for the diagnosis of PTE.

**Parameter**		**Sensitivity**	**Specificity**	**Accuracy**	**PPV**	**NPV**	**k**	**AUC**
Degree of contrast material backwash	No reflux	34.21	29.14	30.84	19.55	46.81	0.27	0.32
	Reflux traces into the IVC only	22.37	82.78	62.56	39.53	67.93	0.61	0.53
	IVC involvement only	21.05	95.36	70.48	69.57	70.59	0.70	0.58
	IVC involvement and involvement of proximal hepatic veins	13.16	94.04	66.96	52.63	68.27	0.66	0.54
	IVC involvement and involvement of the midportion of hepatic veins	7.89	98.68	68.28	75.00	68.04	0.68	0.53
	IVC involvement and involvement of distal hepatic veins	1.32	100.00	66.96	100.00	66.81	0.67	0.51

IV septum deviation	Convex to right ventricle	43.42	7.14	19.13	18.75	20.37	0.16	0.25
	Flattened septum	46.05	94.16	78.26	79.55	77.96	0.77	0.70
	Convex to left ventricle	10.53	98.70	69.57	80.00	69.09	0.69	0.55

Increased RV/LV ratio	Not increased	69.33	7.50	27.23	26.00	34.29	0.25	0.38
	Increased	30.67	92.5	72.77	65.71	74.00	0.72	0.62

Pulmonary artery obstruction index	PAOI < 16	57.89	0.00	18.57	21.46	0.00	0.16	0.29
	PAOI ≥ 16	42.11	100.00	81.43	100.00	78.54	0.81	0.71

Pulmonary infraction	No	1.32	4.97	3.80	0.65	9.64	0.01	0.03
	Unifocal	17.11	96.27	70.89	68.42	71.10	0.70	0.57
	Multifocal	81.58	98.76	93.25	96.88	91.91	0.93	0.90

**Table 4:** The sensitivity, specificity, accuracy, PPV, NPV, and AUC indices of continuous variables of CTPA parameters for the diagnosis of PTE.

**Parameter**	**Threshold**	**Accuracy**	**Sensitivity**	**Specificity**	**PPV**	**NPV**	**AUC**
Ascending aorta diameter	32.88	48.47	53.33	46.10	32.52	66.98	0.45
Azygos vein diameter	9.45	62.83	62.67	62.91	45.63	77.24	0.61
Coronary sinus diameter	9.82	69.52	67.74	70.40	53.16	81.48	0.68
CT obstruction score	1	96.10	98.68	94.84	90.36	99.32	0.96
Pulmonary artery diameter	27.9	59.13	61.84	57.79	41.96	75.42	0.57
RV/LV diameter ratio	0.81	62.13	57.33	64.38	43.00	76.30	0.60

## Data Availability

The data that support the findings of this study are available on request from the corresponding author. The data are not publicly available due to privacy or ethical restrictions.
